# Predicting the Recurrence of Sporadic Odontogenic Keratocyst Using Whole‐Slide Histopathology Images With the Hybrid Encoder Iterative Attention Convolution Model

**DOI:** 10.1002/cre2.70184

**Published:** 2025-07-23

**Authors:** Samahit Mohanty, Divya Biligere Shivanna, Roopa S. Rao, Madhusudan Astekar

**Affiliations:** ^1^ Department of Computer Science and Engineering, Faculty of Engineering and Technology M.S. Ramaiah University of Applied Sciences Bangalore India; ^2^ Department of Oral Pathology, Faculty of Dental Sciences M.S. Ramaiah University of Applied Sciences Bangalore India; ^3^ Department of Oral and Maxillofacial Pathology, Institute of Dental Sciences Bareilly International University Bareilly Uttar Pradesh India

**Keywords:** histopathology, Hybrid Encoder Iterative Attention Convolution (HEIAC) model, odontogenic keratocysts, recurrence prediction, whole‐slide images

## Abstract

**Objectives:**

Odontogenic keratocysts (OKCs) are challenging due to their aggressiveness and high recurrence rates, complicating decision‐making for clinicians and pathologists. Despite efforts to identify predictive characteristics, management remains challenging. The study aims to design a reliable artificial intelligence model to enhance predictive models and distinguish between recurrent and nonrecurrent whole‐slide images of OKCs.

**Material and Methods:**

84 OKC cases were selected for this study, including 29 whole slide images (WSIs) of recurrent OKCs and 35 WSIs of non‐recurrent OKCs for model development. The model was evaluated using 14 non‐recurrent and 6 recurrent cases. The proposed Hybrid Encoder Iterative Attention Convolution (HEIAC) model integrates the strengths of three fundamental components: an encoder, an attention mechanism, and convolutional layers to classify images effectively. The encoder learns to extract useful features, resulting in more meaningful representations that capture the underlying structure of the image data. Iterative attention enables the model to capture intricate details and subtle patterns that may be crucial for accurate image classification. Convolutional layers are designed to learn hierarchical representations of image features automatically. This model harnesses the capabilities of each component to achieve robust and accurate image classification.

**Results:**

The proposed HEIAC model attained 0.98 testing accuracy and exhibits superior performance across the majority of evaluation metrics, achieving 96% recall, 100% precision, a 97% F1‐score, and a perfect AUC of 1.0, and used 96% fewer trainable parameters than the standard vision transformer.

**Conclusions:**

This approach improves predictive models for distinguishing recurrent and non‐recurrent OKCs.

## 1 Introduction

Histopathological whole‐slide image (WSI) is an essential tool for oral cyst diagnosis and therapy, serving as the gold standard for pathological diagnosis. Traditional machine learning and deep learning advancements have demonstrated their value in numerous clinical applications, leveraging WSI automation and delivering accuracy through customized models, thereby providing a direction for pathologists (Bera et al. 2019; Niazi et al. 2019; Zarella et al. 2019). Various traditional machine learning preprocessing methods are used to process inputs. In many cases, the conventional algorithm yields promising results in the diagnostic domain (Yıldız et al. 2024; Yetiş et al. 2024). These tasks include identifying lymph node metastases, subtyping cancer, and determining prognosis. The deep learning research committee has given less attention to the diagnosis and prognosis of oral cysts, resulting in a limited number of publications on the subject (Mercan et al. 2018; Zhang et al. 2022).

Odontogenic keratocysts (OKCs) attract attention as common jaw cysts, characterized by their aggressiveness and tendency to recur. Managing OKC presents challenges due to the high recurrence rates and varied treatment options. Decision‐making relies on cyst size, recurrence risk, and cortical perforation, which is evident in X‐rays. Treatment ranges from conservative (marsupialization) to radical approaches (jaw resection) (Macdonald‐Jankowski 2010; Menon 2015). Some recommend Carnoy's solution (CS) during enucleation to minimize recurrence rates, yet the optimal approach remains disputed (Cakur et al. 2008), with reported recurrence rates ranging widely from 10% to 60% (Walsh et al. 2022; Díaz‐Belenguer et al. 2016). Top‐of‐form theories exist regarding the recurrence of OKCs, such as incomplete removal of the cyst lining, growth from satellite cysts, or remnants of odontogenic epithelial rests (Woolgar et al. 1987). Enucleating OKC intact reduces recurrence compared to numerous pieces (Forssell et al. 1988). OKCs with infection, fistula, or bony perforation have higher recurrence rates (Borghesi et al. 2018). Multilocular cysts recur more often than unilocular ones. Recurrence may not solely depend on surgical technique but also on the inherent biological nature of the lesion and the expression of proliferative markers (González‐Alva et al. 2008; Kuroyanagi et al. 2009; Gelețu et al. 2023).

Classifying recurrent and non‐recurrent OKC images posed challenges due to their diverse biological patterns and spatial positioning variations. Automating OKC recurrence prediction improves patient care by enabling early detection and intervention. Previous studies, such as those by Augustine et al. (2021), have identified potential histological features associated with recurrence. However, automation attempts by Rao et al. (2022) and Mohanty et al. (2023) were limited by small sample sizes. Larger‐scale studies are crucial to validate these findings and enhance predictive models. This study aims to validate and provide clinicians with a robust tool to distinguish between recurrent and nonrecurrent OKCs by developing a reliable AI model using a larger sample size. These advancements promise better clinical decision‐making and improved patient outcomes in OKC treatment.

Cystic jaw lesions, particularly OKCs, pose significant challenges for clinicians in diagnosis and surgery due to their aggressive nature and high recurrence rates. Current management options are debated, and aggressive surgical treatments can have psychological and physical impacts on patients, including loss of bone and teeth and esthetic concerns. Therefore, careful patient selection and accurate diagnosis of recurrent histopathological features are crucial for guiding tailored treatment before surgery.

## 2 Methods

Two hospitals collaborated to manage patients over extended periods, utilizing H&E‐stained incisional biopsy specimens for automation. They include the M.S. Ramaiah University of Applied Sciences (MSRUAS) in Bangalore, the Institute of Dental Sciences, Bareilly International University (BIU) in Bareilly, Uttar Pradesh. The MSRUAS (EC‐2021/F/058) and BIU (IEC/292/2024/04) granted ethics clearance for the archiving and exploring of slides.

Eighty‐four clinically documented cases of sporadic OKCs were retrieved from two centers, including archived H&E‐stained slides from FFPE blocks. These slides were digitized into whole‐slide images (WSIs) using the Morphle Labs Whole Slide Scanner Model‐Index, equipped with a 40x Infinity Plan Achromat lens and single‐slide loading capacity. Of the 84 cases, 64 (29 recurrent, 35 non‐recurrent) were used to develop a deep learning model for recurrence prediction. 20 WSI were used to test the model. Here, the data split was 75% for creating the model and 25% for testing.

After annotation, an automated system generated tile images of 2048 × 2048 pixels using OpenSlide and Deep Zoom Generator. White tiles were excluded based on entropy and variance calculations. Tiles with low entropy or variance, indicating uniform or low information content, were labeled as empty. The pathologist's review confirmed the accurate labeling of each tile as recurrent or non‐recurrent. Preprocessing utilized the highest zoom‐level slides to generate small images of 2048 pixels, referred to as “Tiled Images.” A total of 84 WSIs were included in the study, with 64 WSIs used for model development and 20 WSIs reserved for testing. The 64 WSIs used in training were tiled, and 111,600 white tiles were discarded. Additionally, 1200 tiles were removed due to blurriness, and 1100 tiles were excluded because of overstaining or the presence of artifacts. An experienced pathologist performed annotation, and to minimize subjectivity and interobserver bias, a second pathologist involved in the study was consulted, resulting in a consensus between them. In conclusion, 466 recurrent and 524 non‐recurrent tiled images were chosen for model development. The data set was experimented with using standard CNNs, pre‐trained models, and vision transformer algorithms for Rec and Non‐Rec OKC WSI classification. The proposed attention‐based model and the standard vision transformer model were compared. The training, validation, and test images were split into a 70:10:20 ratio from the preprocessed tiled data set. These are tiled images of size 2048. Table 1 has all the details of the data set.

**Table 1:** Data set description.

The total number of WSI	Recurring OKC	Non‐recurring OKC
64 cases from MS Ramaiah University Each case results in one WSI	29 WSI used for training and validation of the proposed pipeline along with HEIAC	35 WSI used for training and validation of the proposed pipeline along with HEIAC
20 cases from the Institute of Dental Sciences, Bareilly Each case results in one WSI	6 WSI used for testing the proposed complete pipeline along with HEIAC	14 WSI used for testing the proposed complete pipeline along with HEIAC
For building the HEIAC model 64 WSI, the WSI was tiled to 2048 × 2048, and white, over/under stained, blurred tiles were removed		
Number of tiles used for building the HEIAC model	466	524
Total number of tiles	990	
Tiles used for training and validation of the HEIAC model	366	424
Tiles used for testing the HEIAC model alone	100	100

Table 2 describes the details of the hyperparameters used in the proposed model.

**Table 2:** Hyperparameters used in the models.

Component	ViT	ABIAS	HEIAC
Learning rate	0.0001		
Batch size	20		15
Epochs	25		
Weight decay	0.001		
Dropout rate	0.1		0.5
Optimizer	Adam		
Loss function	Sparse categorical cross‐entropy		Binary‐cross‐entropy
Activation function	Gaussian error linear unit		

Unlike model parameters, hyperparameters are predefined external settings or configurations that must be determined before training. All models use a consistent learning rate of 0.0001. Batch sizes range from 15 to 20, with HEIAC utilizing a smaller size (15) than ViT and ABIAS (20). All models employ the same number of epochs (25). All models use the same weight decay rate of 0.001. ViT utilizes a dropout rate of 0.1, whereas ABIAS employs a dropout rate of 0.5. A lower dropout rate, such as 0.1. The model retains more information during training iterations, dropping fewer neurons and potentially accelerating learning. The optimization algorithm updates the model's parameters during training, using the Adam optimizer for all models. The proposed Hybrid Encoder Iterative Attention Convolution (HEIAC) model, which integrates the encoder, attention mechanism, and convolution layers, attained higher accuracy. By leveraging these components, HEIAC efficiently categorizes tiles, offering reliable and effective image classification. The encoder component extracts intricate details and critical features from input images, compressing them into a meaningful representation. This reduces data dimensionality and captures essential visual information necessary for classification. Augmenting the encoder, the attention mechanism sharpens the model's focus on pertinent regions within the encoded representation. Using iterative attention, the model dynamically adjusts its focus, extracting discriminative features from various parts of the image. This iterative refinement enables the model to capture fine‐grained details, effectively addressing the complex visual patterns and variations in the images. Additionally, convolution layers are pivotal in extracting features and learning hierarchical representations. Convolution layers detect important patterns, edges, and textures across input images at different scales using convolution operations. This hierarchical feature learning enables the model to grasp local and global visual cues, which is essential for precise classification. The HEIAC model seamlessly integrates encoder‐based feature extraction, attention‐driven refinement, and convolutional feature learning, thereby enhancing its ability to accurately label images across diverse datasets and real‐life situations.

**Figure 1:**
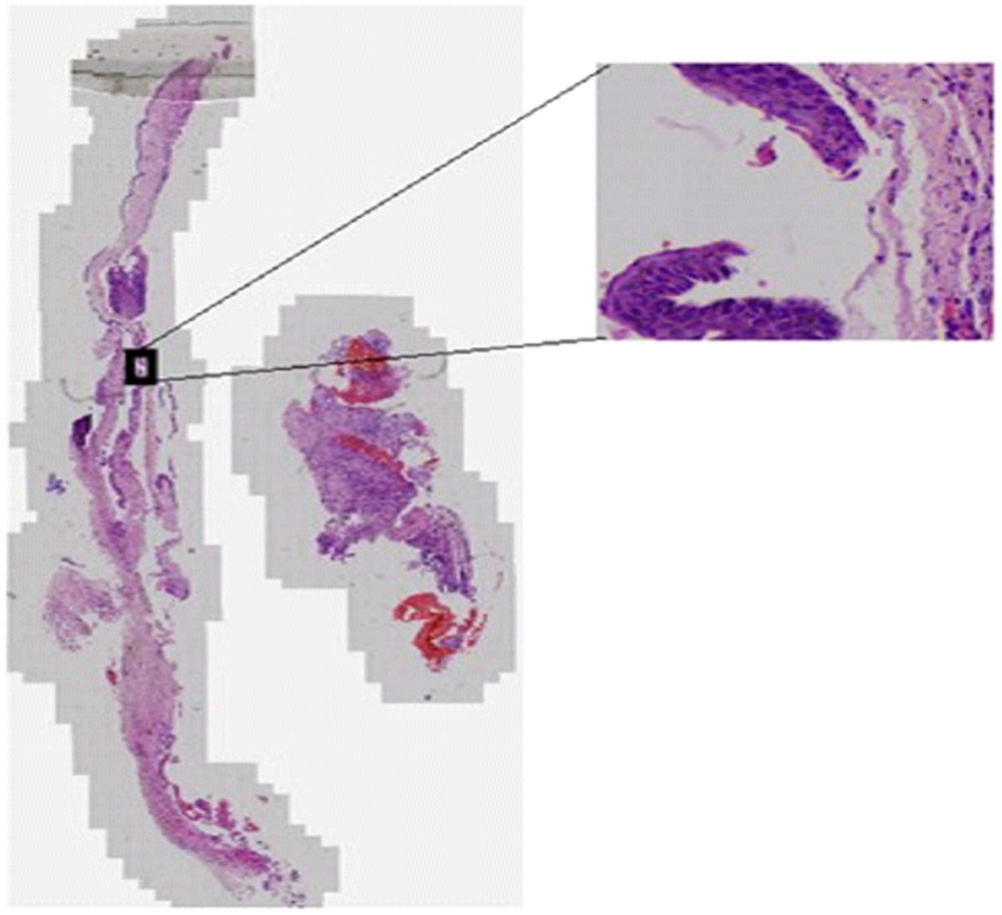
Whole slide image (WSI) in 1.3× magnification of recurrent OKC.

**Figure 2:**
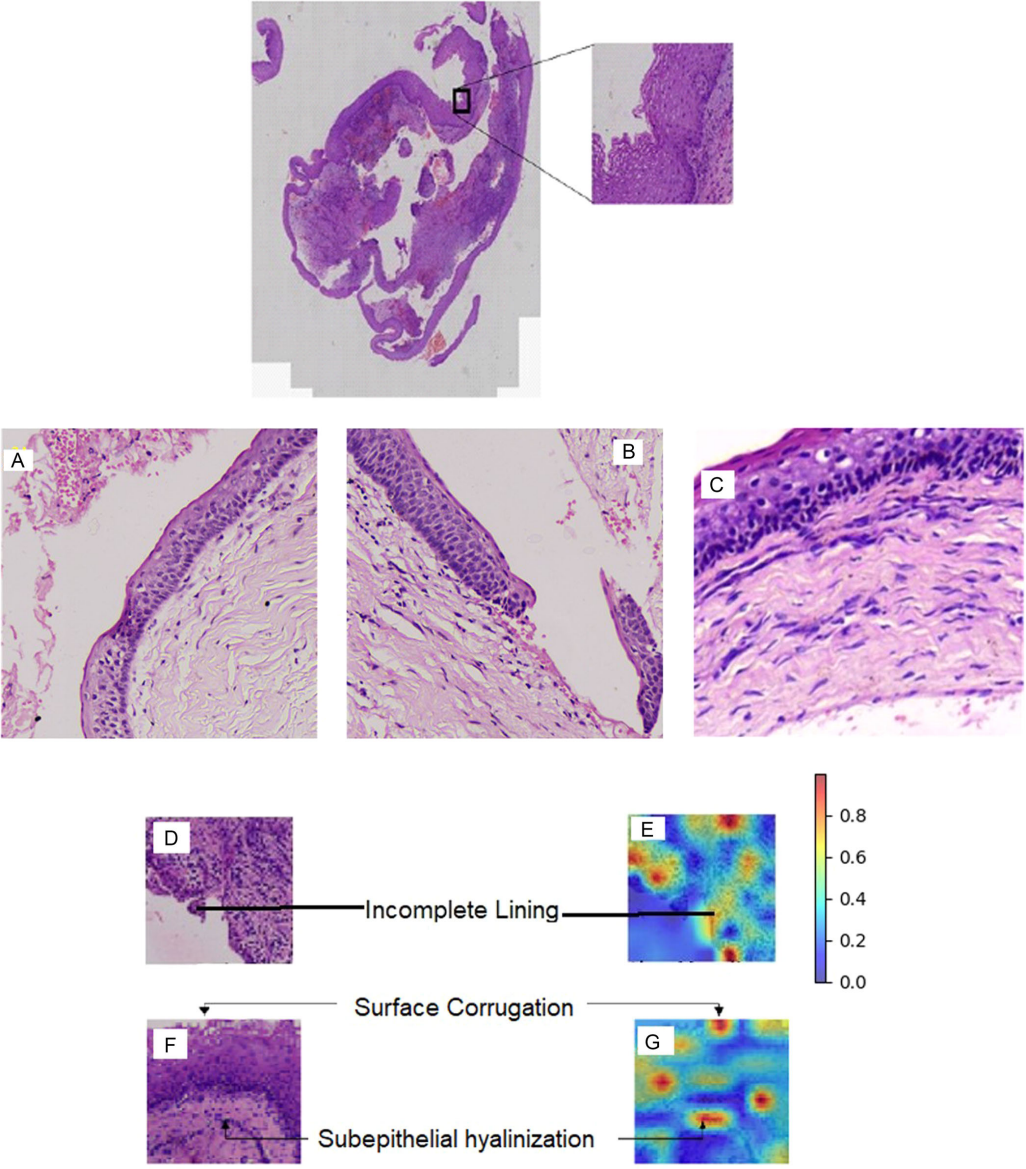
(Top) Whole slide image (WSI) in 1.3 × magnification of non‐recurrent OKC. (A) H&E‐stained section at 40 × magnification, showing surface corrugations and sub‐epithelial hyalinization, characteristic of recurrent odontogenic keratocyst. (B) Breach in the surface epithelium at 40 × magnification, contributing to recurrence in odontogenic keratocyst. (C) H&E‐stained section at 40 × magnification shows absence of surface corrugations, sub‐epithelial hyalinization, and breach in the epithelium, favoring non‐recurrent odontogenic keratocyst. (D, F) H&E‐stained section at 40x magnification shows the presence of incomplete epithelial lining surface corrugations and sub‐epithelial hyalinization, favoring non‐recurrent odontogenic keratocyst. (E, G) The heat map of the corresponding images generated by the attention layers.

**Interpretability Analysis:** Key histopathological features associated with recurrence include subepithelial hyalinization, surface corrugations, and incomplete epithelial lining, which favor recurrent OKC under 40× magnification (Figures 1 and 2A,B).

The H&E‐stained WSI section shows intact epithelial and connective tissue junctions, the absence of sub‐epithelial hyalinization, and surface corrugations, which favor a non‐recurrent OKC under 40× magnification (Figure 2C).

The attention heat map generated by the model reveals that surface corrugations are assigned substantial significance in the decision‐making process, as evidenced by the intense red coloration in the corresponding regions, as shown in Figure 2D,H,G. This visual emphasis indicates that the model attributes higher attention weights to this morphological feature, suggesting its potential diagnostic relevance within the learned representation.

Moreover, the model concurrently highlights incomplete epithelial lining and subepithelial hyalinization as additional key features influencing the classification outcome. The consistent focus on these specific histopathological structures implies that the model has successfully learned to recognize patterns that are traditionally considered important by expert pathologists.

This alignment between the model's attention and domain‐specific morphological indicators underscores the interpretability and clinical relevance of the model's predictions. It also reflects the potential of attention‐based deep learning frameworks to not only achieve high accuracy but also offer meaningful insights into the underlying pathological features driving the classification, thereby enhancing trust and transparency in AI‐assisted diagnostics.

**Figure 3:**
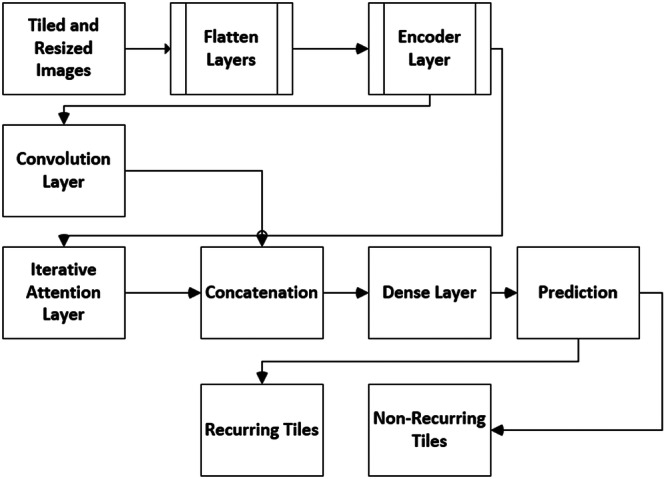
Overall flow of the HEIAC model.

**Figure 4:**
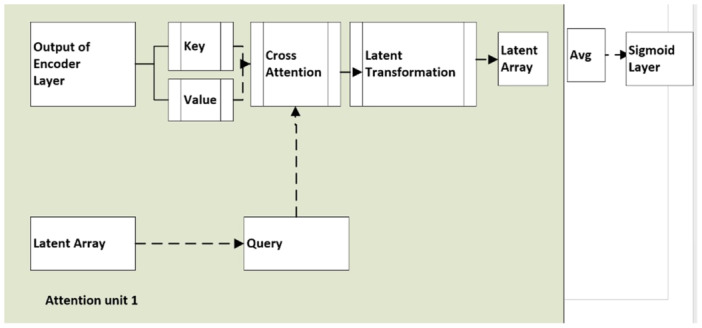
Iterative attention layer in HEIAC.

A logical overall research workflow (Figures 3 and 4) was constructed to determine the number of recurrent and non‐recurrent tiles. Trial and error led to establishing a threshold, which states that the WSI is classified as non‐recurrent if it accounts for more than 20% of the recurrent tiles. The encoder layer takes in the 512 × 512 × 3 tiled and scaled image, flattening it into a 786,432‐element array. Within this layer, a three‐layered unsupervised neural network generates a 12,288‐element feature. This setup is part of an autoencoder designed to use its encoder layers for efficient feature extraction from input data. In this setup, feature extraction happens implicitly during training. As data moves through the three encoder layers, nonlinear transformations are applied, abstracting and refining essential features. This process reduces the data's dimensionality by compressing it into a lower‐dimensional latent space while retaining its critical features. The latent space representation from the third encoder layer condenses the essential features of the input data into a feature vector. These features are learned implicitly during unsupervised training without labeled examples. As a bottleneck layer, the third encoder efficiently captures key features with lower dimensionality than the input data. The Iterative Attention model received these features (Figure 3). To predict if the tile was recurrent or non‐recurrent, the output of the iterative model and convolutional layers were concatenated and sent to the dense layers. The output of the encoder layer was input to generate keys and values. A linear transformation was used to generate a key.

key=key_weight_matrix × Input_features+bias

Similarly, Value was generated by

value=value_weight_matrix × Input_features+bias

Similarly, a query was generated by

query=query_weight_matrix × Input_features+bias

Queries are used to attend over the keys and values.

The model computes attention scores between queries and keys to compute cross‐attention. The attention scores ats_i_ between queries

$${\text{ats}}_{i}=\text{softmax}\;(\text{query ×}\;{\text{key}}_{i}/\text{sqrt}(\text{dimension of the key vector})$$

Finally, the attention scores were used to aggregate values corresponding to the keys that have been attended to:

$$\text{CrossAttention}=\sum {\text{ats}}_{i}\times {\text{Value}}_{i}$$

Iterative attention mechanisms, as shown in Figure 4, empower the model to concentrate on diverse features, extracting intricate details over multiple iterations. This iterative refinement enables the model to capture essential subtle patterns, aiding accurate classification by identifying discriminative features. Moreover, it enhances interpretability and allows more precise feature localization. The model adapts to feature changes by iteratively updating attention weights, resulting in more robust and generalizable classification performance.

The proposed model includes trainable weight matrices and bias vectors, which underwent optimization during training to enable efficient cross‐attention computations. The latent array resides in a “latent space,” a feature space where similar concepts are closer together. This space is designed to be semantically meaningful, enabling the model to undertake diverse downstream tasks, such as classification. Latent arrays allow attention mechanisms to selectively emphasize relevant information in the input sequence while suppressing noise or irrelevant details. This selective attention enhances the model's capacity to comprehend and handle intricate sequences more effectively. With latent arrays, attention mechanisms capture contextual information from the input sequence, allowing the model to comprehend dependencies and relationships between elements. Latent transformations reduce the dimensionality of input data before attention mechanisms, aiming to improve computational efficiency, capture critical features, and promote better learning and generalization.

**Figure 5:**
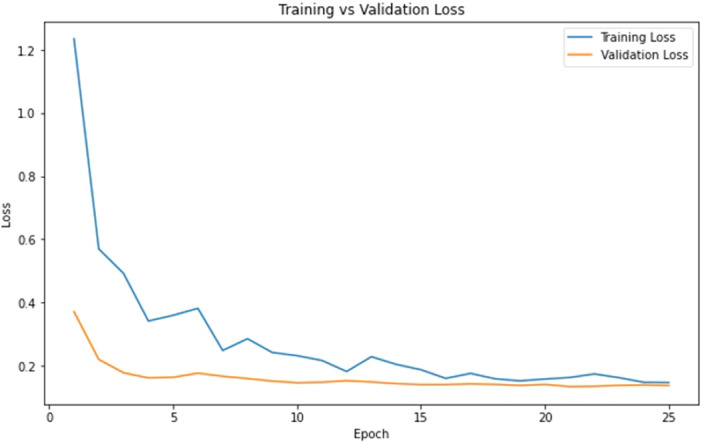
Training and validation graph of HEIAC.

Iterative attention mechanisms expand the conventional approach by performing multiple rounds of attention computation, refining attention weights iteratively through feedback mechanisms. This experiment employed three distinct attention units to identify pertinent information in the input data. The resultant latent arrays generated by these units were then combined through averaging. Finally, the aggregated representation underwent processing through a sigmoid layer to yield the final output. Model validation was done using metrics including Recall, Precision, F1‐Score, AUC, Accuracy, and Total Parameters. All these metrics and their impact are discussed in the results section. Figure 5 provides a training and validation graph. The validation loss also decreases and stabilizes at a low value, indicating excellent generalization. There is no significant divergence from the training loss, indicating that the model is not overfitting.

## 3 Results

Table 3 presents a comparison of results between different models. The table presents a comparative analysis of various deep learning models based on several performance metrics, including Recall, Precision, F1‐Score, AUC, Accuracy, and Total Parameters.

**Table 3:** Overall performance metrics of various experiment models.

Model	Recall	Precision	F1‐Score	AUC	Accuracy	Total parameters
Standard convolutional neural network (CNN)	0.86	0.86	0.84	0.93	0.84	683,329
Vgg19	0.73	0.81	0.73	0.77	0.73	131,585
Vgg16	0.80	0.84	0.80	0.82	0.80	131,585
Inception V3	0.82	0.82	0.82	0.78	0.82	23,901,985
Standard ViT	0.95	0.86	0.90	0.91	0.91	15,488,969
Attention‐based image sequence analyzer	1.00	0.96	0.98	0.98	0.98	8,947,721
HEIAC	0.96	1.0	0.97	1.0	0.98	515974

Table 4 displays the model summary.

**Table 4:** Model architecture summary.

Layer (Type)	Output shape	Param #	Connected to
Input_layer_26 (Input Layer)	(None, 64, 64, 3)	0	—
Conv2d_14 (Conv2D)	(None, 64, 64, 32)	896	Input_layer26[0][0]
Max_pooling2d_6 (MaxPooling2D)	(None, 32, 32, 32)	0	Conv2d_14[0][0]
Conv2d_15 (Conv2D)	(None, 30, 30, 64)	18,496	Max_pooling2d_6[0] [‐
Max_pooling2d_7 (MaxPooling2D)	(None, 15, 15, 64)	0	Conv2d_15[0][0]
Conv2d_16 (Conv2D)	(None, 13, 13, 128)	73,856	Max_pooling2d_7[0] [‐
Max_pooling2d_8 (MaxPooling2D)	(None, 6, 6, 128)	0	Conv2d_16[0][0]
Conv2d_17 (Conv2D)	(None, 4, 4, 64)	73,792	Max_pooling2d_8[0] [‐
Global_Average_pool_(GlobalAveragePooling)	(None, 4, 4, 64)	0	Conv2d_17[0][0]
Conv2d_17 (Conv2D)	(None, 64)	0	Conv2d_17[0][0]
Perceiver_1 (Perceiver)	(None, 2)	348,867	Input_layer26[0][0]
Concatenate_1 (Concatenate)	(None, 66)	0	Global_average_pool_Perceiver_1[0][0]
Dense_63	[None,1]	67	Concatenate_1[0][0]
Total parameters: 515,974 (1.97 MB) Trainable parameters: 515,974 (1.97 MB) Non‐trainable parameters: 0 (0.0 B)			

**Figure 6:**
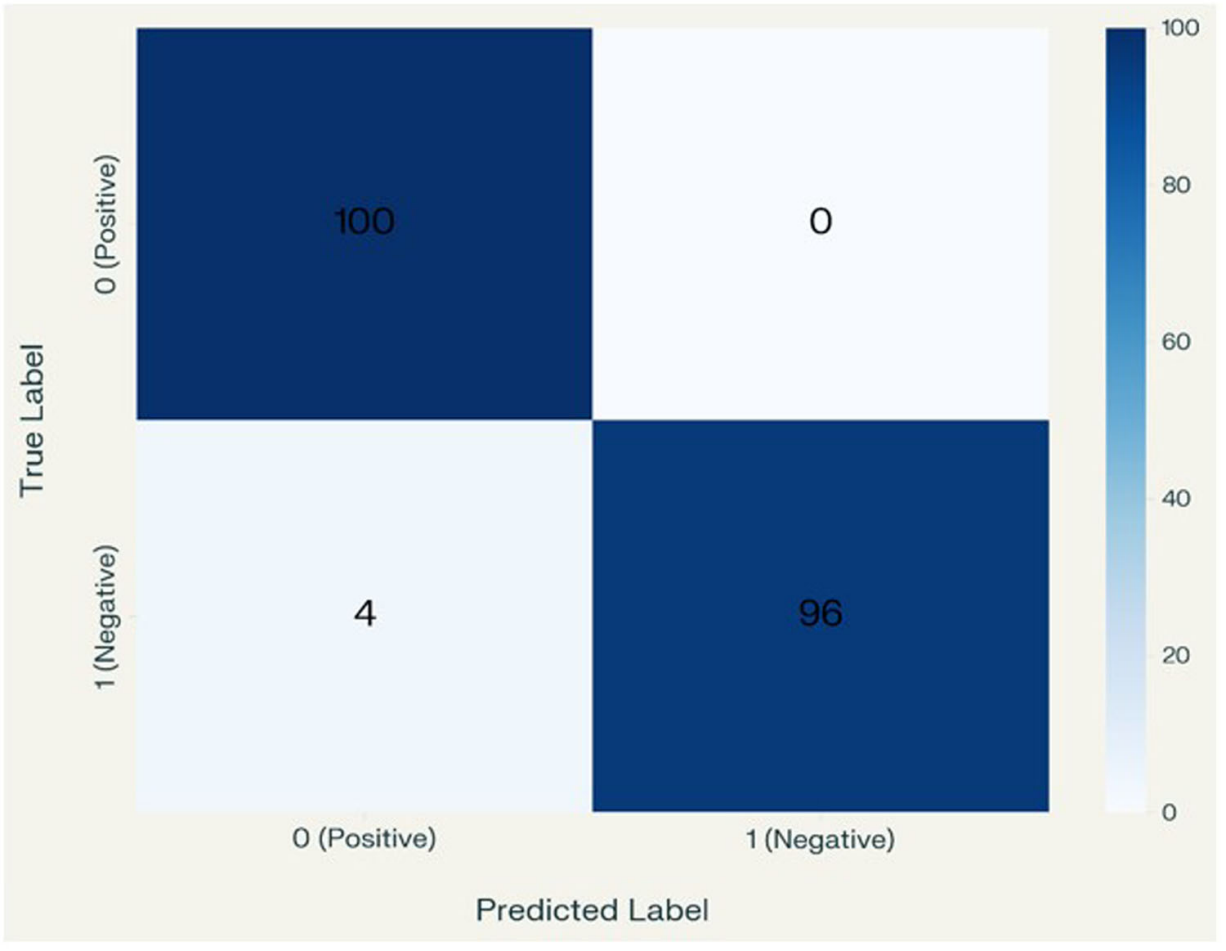
Confusion matrix.

**Figure 7:**
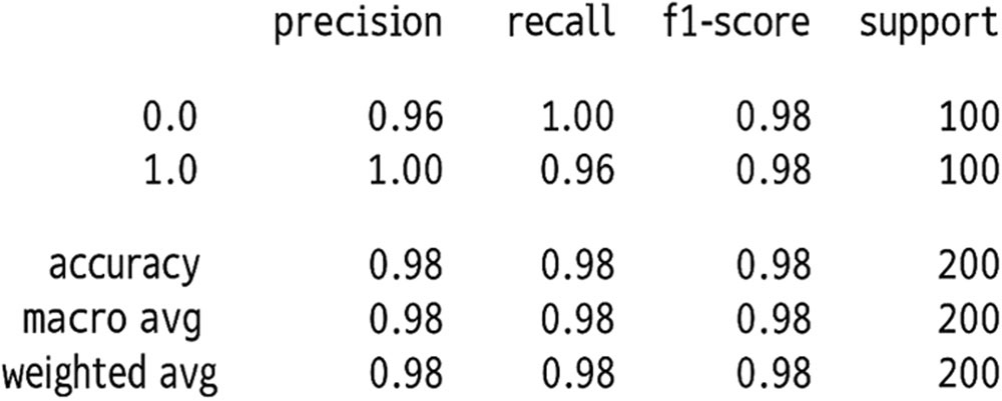
Performance report.

**Figure 8:**
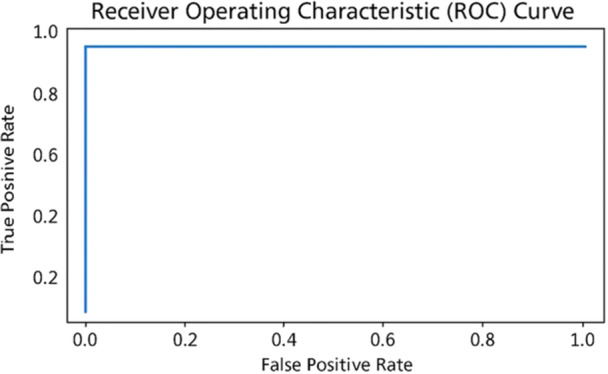
ROC curve.

The HEIAC model demonstrates the highest performance across most metrics, achieving 96% recall, 100% precision, a 97% F1‐score, and a perfect 1.0 AUC. This suggests that it has the highest ability to balance false positives and false negatives while achieving a high accuracy rate of 98%. Yet, its precision (1.0) with slightly lower recall (0.96) suggests a potential bias toward positive predictions. Attention‐Based Image Sequence Analyzer also performs exceptionally well, achieving 100% recall, 96% precision, 98% F1‐score, and 98% accuracy. A standard Convolutional Neural Network (CNN) performs moderately well, achieving 86% recall and precision, 84% F1‐score, and 84% accuracy. It has a relatively small number of parameters (683,329), indicating that it is computationally efficient. VGG19 has the lowest recall (73%) and F1‐score (73%), suggesting that it is less effective at correctly identifying positive cases. VGG16 outperforms VGG19 with improved recall (80%) and F1‐score (80%) but still lags behind other models. Both have the same number of parameters (131,585), making them lightweight but less accurate. Inception V3 Performance demonstrates a balanced performance with 82% recall, precision, and accuracy; however, its AUC (0.78) is lower than that of most models. However, it has a vast number of parameters (23.9 M), making it computationally expensive compared to others with similar performance. Standard ViT achieves high scores, including 95% recall, 86% precision, 90% F1‐score, and 91% accuracy, demonstrating its effectiveness in image classification. However, it has a large number of parameters (15.4 M), indicating higher computational demands. Figure 6 displays the resulting confusion matrix, receiver operating characteristic (ROC) curve, and performance report. The study developed a pipeline system for precisely detecting recurrent and non‐recurrent OKC WSIs using an attention‐based image sequence analyzer (ABISA) [24]. It achieved 98% accuracy with 8,947,721 trainable parameters; however, in the current experiment, 98% accuracy was attained with only 515,974 trainable parameters. Table 5 presents a comparative analysis of various metrics obtained during the experiment. Figure 6 shows the confusion matrix, Figure 7 shows the classification report, and Figure 8 shows the ROC curve of the model.

**Table 5:** Model comparative analysis.

Model	Strengths	Weaknesses
HEIAC	Best precision (1.0), AUC (1.0), accuracy (98%), less number of parameters	None (Best overall)
Attention‐based analyzer	Highest recall (1.0), strong F1‐score, and AUC (0.98)	Slightly lower precision (0.96)
Standard ViT	High recall (0.95), balanced precision, F1‐score, and accuracy	Large model size (15.4 M parameters)
Inception V3	Decent accuracy (0.82) and balance	Very large parameters (23.9 M), lower AUC (0.78)
VGG19	Small model size (131 K parameters)	Lowest recall and F1‐score (0.73)
VGG16	Better than VGG19 in recall (0.80)	Lower AUC (0.82) and accuracy (0.80)
Standard CNN	Balanced performance, small model size (683 K)	Lower than transformer‐based models

### 3.1 Pipeline Result (Entire Whole Slide Testing)

**Figure 9:**
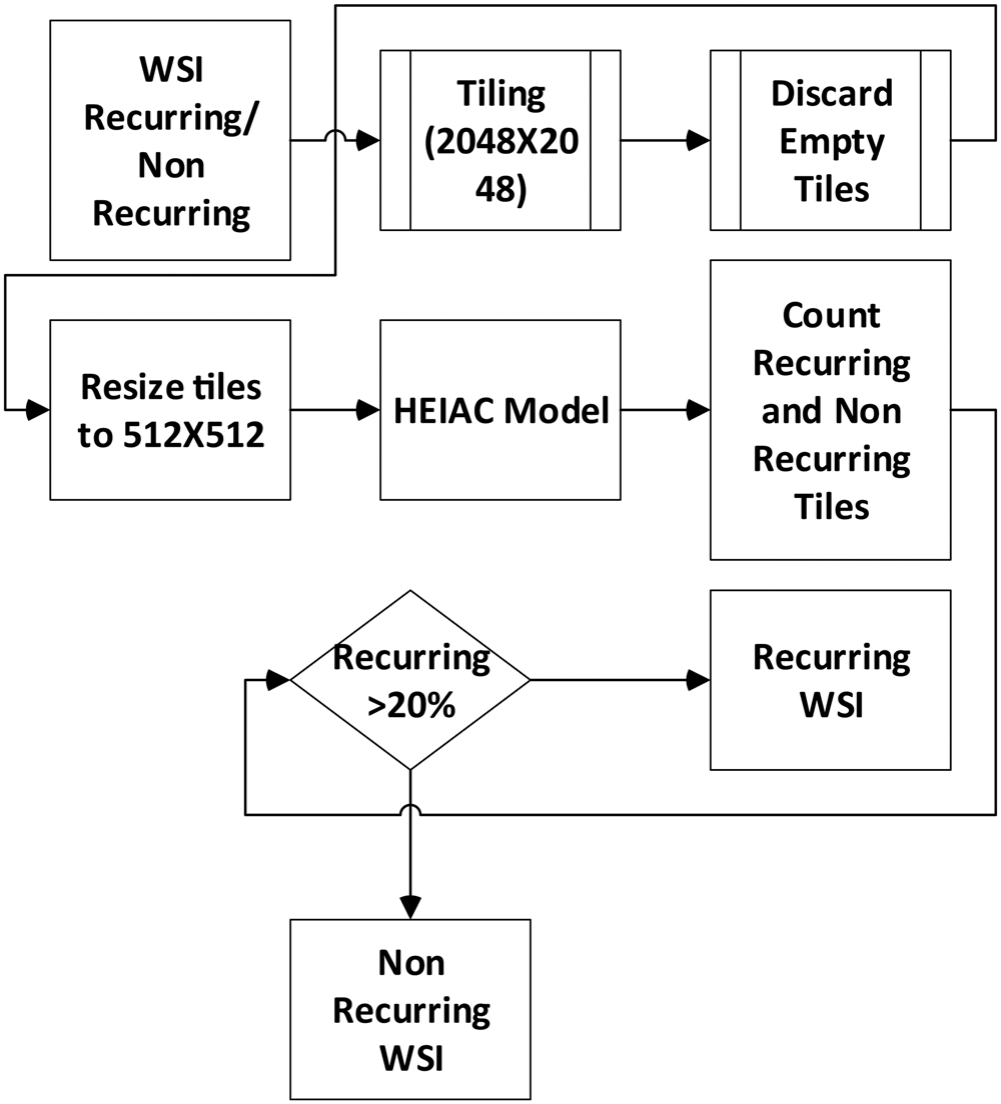
Overall flow of the pipeline.

Twenty H&E‐stained WSIs were tested using the pipeline, comprising 14 non‐recurrent and six recurrent cases from the Institute of Dental Sciences, Bareilly. The pipeline correctly identified all 14 non‐recurrent cases. However, one of the six recurrent cases was misclassified as non‐recurrent. The pipeline was developed using a data set of 64 WSIs from MS Ramaiah University of Applied Sciences, Bangalore, which included 29 recurrent and 35 non‐recurrent cases. These WSIs were not used in model training or validation. The framework's predictive accuracy was independently assessed in the presence of a pathologist, demonstrating its clinical utility in classifying recurrent and non‐recurrent OKCs. Figure 9 illustrates the process flow and lists the sample WSIs used in pipeline experiments.

## 4 Conclusion

The HEIAC model was developed to effectively capture spatial and sequential patterns in both recurrent and non‐recurrent OKC images. It integrates attention mechanisms to refine feature representations iteratively, concentrating on relevant areas of the input data. Convolutional layers are employed to learn hierarchical features from images, which is crucial for medical image analysis. The HEIAC model was explicitly used to predict the recurrence of OKCs after treatment. The HEIAC model, with its hybrid encoder, iterative attention, and convolution layers, achieves remarkable accuracy in classifying recurrent and non‐recurrent OKC WSIs. Combining these components effectively and efficiently categorizes images, ensuring robust classification crucial for surgical planning. The data set excludes blurry or inadequately stained images, enhancing its reliability. This study could assist clinicians in proactive surgical management through automated histopathology reports and holds promise for extending to other cancers that rely on WSI analysis.

## Author Contributions

**Samahit Mohanty:** algorithm design and implementation. **Divya Biligere Shivanna:** research methodology and research guide. **Roopa S. Rao:** conceptualization and pathologist. **Madhusudan Astekar:** a pathologist who helped in validating annotations. All authors have read and agreed to the published version of the manuscript.

## Ethics Statement

This study was conducted at MS Ramaiah Dental College, MS Ramaiah University of Applied Sciences. Ethics approval was waived due to the study's retrospective nature, and the collected slides were anonymized and encoded to eliminate any patient identifiers.

## Conflicts of Interest

The authors declare no conflicts of interest.

## Data Availability

Restrictions apply to the availability of these data. Data was obtained from the mentioned centers and is available from the authors with permission.
